# Special Issue “Diagnostic Biomarkers in Prostate Cancer 2020”

**DOI:** 10.3390/diagnostics11030505

**Published:** 2021-03-12

**Authors:** Jochen Neuhaus

**Affiliations:** Research Laboratory, Department of Urology, University of Leipzig, 04103 Leipzig, Germany; jochen.neuhaus@medizin.uni-leipzig.de

The search for novel prostate cancer biomarkers is one of the major topics in recent urologic research. Given the still unsatisfactory performance of the available diagnostic biomarkers in the detection of significant prostate cancer cases and the lack of prognostic and predictive biomarkers with high specificity, there is still an urgent need for new biomarkers. This special issue presents nine original research papers and two reviews elucidating novel developments in prostate cancer biomarkers. Interestingly, two major topics were in focus, microRNAs (miRNAs) as biomarkers [1,2,3,4] and the potential role of oxidative stress and antioxidant status in prostate cancer [5,6].

The fast-growing field of miRNA biomarkers in liquid biopsies reflects the high expectations raised in the implementation of miRNA diagnostics. Fredsøe and colleagues analyzed 92 circulating miRNAs in plasma samples of 753 patients and found distinct regulation of miRNAs in patients with benign prostatic hyperplasia (BPH), localized prostate cancer, and advanced prostate cancer with large overlaps between the groups [2]. The individual 92 miRNAs were only weakly associated with the prognosis of PCa. However, a 4-miRNA ratio model showed decent performance in the prediction of positive transrectal ultrasound guided biopsies, and in combination with prostate specific antigen (PSA), the accuracy was higher than PSA alone, indicating a possible advantage in the pre-selection of patients for prostate biopsy.

Using urine and urinary cells, respectively, is the second major approach in minimally invasive liquid biopsies for PCa detection. Borkowetz and colleagues used urinary sediments to analyze a 12-miRNA panel by qPCR [1]. Two miRNAs were able to predict tumor in patients suspected for PCa with higher accuracy, sensitivity and specificity than PSA. The diagnostic performance was comparable to PSA-density, and the combination of PSA-density with the two most promising miRNAs further improved the accuracy. Even more distinct was this finding in patients with PSA ≤ 10 ng/mL, suggesting a potential value of miRNA diagnostics in prediction of positive biopsies, thus reducing the need of prostate biopsies.

Successful implementation in routine clinical use requires standardization of miRNA measurements. Konoshenko and coworkers evaluated the diagnostic potential of ratios constructed from a panel of 12 cell-free miRNAs, using urine extracellular vesicles, clarified urine, and plasma. Eight miRNAs combined into six ratios showed maximum stability and 97.5% accuracy in separating PCa patients from the control group (healthy donors + BPH) [3]. In a second paper, the authors investigated the potential use of the previously described 12 cell-free miRNAs as predictive biomarkers to monitor the therapeutical efficacy of radical prostatectomy (RPE). Again, urine extracellular vesicles proved the most stable source of miRNA biomarkers, and three candidate ratios were able to monitor the therapeutical effect of RPE. The authors conclude that sequential tests every few months after RPE and the comparison to the miRNA status at discharge can be used to monitor the patient’s recovery and the state of the tumor [3]. The work of Konoshenko and colleagues is notable in two regards: (i) presenting a stable diagnostic tool for improved PCa detection (100% sensitivity, 100% specificity) and (ii) demonstrating the potential use of a subset for follow-up monitoring of the patients. Both studies warrant validation in larger cohorts, which we anticipate with great interest.

Blood and urine are also profitable sources of metabolites, which have recently been used in numerous disease states. Yang and colleagues conducted a study searching for urine metabolite biomarkers for the detection of PCa. They found twenty differentially expressed urine metabolites in a cohort of 50 prostate cancer patients compared to non-cancerous individuals [7]. The combination of solely three metabolites, representing alterations in Glycine, Serine, and Threonine metabolism (KEGG database pathway), was able to identify PCa patients with 77% accuracy at 80% sensitivity and 64% specificity. Furthermore, those metabolites could separate significant PCa (Gleason score ≥ 7) from indolent PCa (GS 6), which confirms urine metabolomics as a promising diagnostic tool in PCa.

While liquid biopsies are an upcoming promising tool of non-invasive PCa diagnostics, verification of biomarkers in tissues is mandatory to prove PCa being the primary cause of the alterations in liquid biopsy biomarkers. Latosinska and colleagues present a proteomic study comparing the proteome of PCa tissue with benign prostatic hyperplasia (BPH), a common co-morbidity especially in aged patients [8]. They detected 145 differently abundant proteins by liquid chromatography coupled to tandem mass spectrometry (LC-MS/MS). In silico analysis revealed a correlation of 21 of those proteins with PCa progression and a central role of Myc proto-oncogene.

Patients with GS ≤ 6, i.e., at low risk, are eligible for active surveillance. Yu and colleagues asked whether multiparametric MRI (mpMRI) can be a substitute or supplement of traditional tools as PSA, digital rectal examination, and transrectal ultrasound-guided biopsy (TRUS) [9]. They retrospectively analyzed the follow-ups of 355 men under clinical consideration for or already in active surveillance. They found that mpMRI identified otherwise undiagnosed PCa during active surveillance leading to upgrading in 22% of the patients. These results speak in favor for the implementation of mpMRI in active surveillance.

Two papers in this special issue elucidate oxidative stress related enzymes and peptides as potential biomarkers in PCa. As shown by Veljković and colleagues, xanthine oxidase (XO) activity was significantly higher in tumor tissue compared to heathy controls and strongly correlated with serum PSA levels [5]. The authors conclude that XO may be involved in carcinogenesis of PCa and XO inhibitors could be useful in adjuvant therapy. In line with this notion, Shukla and colleagues report oxidative DNA damage and reduced anti-oxidative capacity in high-risk patients compared to healthy controls [6]. 

Finally, two reviews provide updates on prognostic and predictive omics-derived biomarkers for therapy of advanced PCa and especially of serum biomarkers in metastatic disease [10,11]. In their systematic review, Frantzi and colleagues analyzed 56 (out of 3035) articles for the performance and clinical validity of prognostic and predictive biomarkers to give a perspective on personalized PCa treatment. They conclude that currently available data from mostly explorative studies well support the use of various omics-derived biomarkers in the management of advanced PCa, but larger clinical studies, necessary for routine clinical implementation, are missing because of the lack of funding opportunities.

Saxby and colleagues focused on serum biomarkers (miRNA, androgen receptor variants, bone metabolism, neuroendocrine, metabolite), which can be used in conjunction with PSA as prognostic or predictive biomarkers for metastatic PCa. Forty-three were analyzed and revealed numerous biomarkers with potential value for identifying patients with advanced PCa and of value for the development of targeted treatment strategies. However, while basic data are available, clinical validation in prospective trials is urgently needed to bring them to routine use in clinical practice.

This special issue elucidates some important aspects of PCa biomarker development and their potential clinical benefit and highlights advanced techniques of biomarker discovery. The future of those biomarkers strongly depends on prospective multicenter clinical diagnostic studies, which hopefully will find the support of the major funding agencies.

## References

[1] Borkowetz A., Lohse-Fischer A., Scholze J., Lotzkat U., Thomas C., Wirth M.P., Fuessel S., Erdmann K. (2020). Evaluation of MicroRNAs as Non-Invasive Diagnostic Markers in Urinary Cells from Patients with Suspected Prostate Cancer. Diagnostics.

[2] Fredsøe J., Rasmussen A.K.I., Mouritzen P., Bjerre M.T., Østergren P., Fode M., Borre M., Sørensen K.D. (2020). Profiling of Circulating microRNAs in Prostate Cancer Reveals Diagnostic Biomarker Potential. Diagnostics.

[3] Konoshenko M.Y., Bryzgunova O.E., Lekchnov E.A., Amelina E.V., Yarmoschuk S.V., Pak S.V., Laktionov P.P. (2020). The Influence of Radical Prostatectomy on the Expression of Cell-Free MiRNA. Diagnostics.

[4] Konoshenko M.Y., Lekchnov E.A., Bryzgunova O.E., Zaporozhchenko I.A., Yarmoschuk S.V., Pashkovskaya O.A., Pak S.V., Laktionov P.P. (2020). The Panel of 12 Cell-Free MicroRNAs as Potential Biomarkers in Prostate Neoplasms. Diagnostics.

[5] Veljković A., Hadži-Dokić J., Sokolović D., Bašić D., Veličković-Janković L., Stojanović M., Popović D., Kocić G. (2020). Xanthine Oxidase/Dehydrogenase Activity as a Source of Oxidative Stress in Prostate Cancer Tissue. Diagnostics.

[6] Shukla S., Srivastava J.K., Shankar E., Kanwal R., Nawab A., Sharma H., Bhaskaran N., Ponsky L.E., Fu P., MacLennan G.T. (2020). Oxidative Stress and Antioxidant Status in High-Risk Prostate Cancer Subjects. Diagnostics.

[7] Yang B., Zhang C., Cheng S., Li G., Griebel J., Neuhaus J. (2021). Novel Metabolic Signatures of Prostate Cancer Revealed by 1H-NMR Metabolomics of Urine. Diagnostics.

[8] Latosinska A., Davalieva K., Makridakis M., Mullen W., Schanstra J.P., Vlahou A., Mischak H., Frantzi M. (2020). Molecular Changes in Tissue Proteome during Prostate Cancer Development: Proof-of-Principle Investigation. Diagnostics.

[9] Yu J., Fulcher A.S., Winks S., Turner M.A., Behl W., Ware A.L., Mukhopadhyay N.D., Kim C., Jackson C., Bajaj H.S. (2020). Utilization of Multiparametric MRI of Prostate in Patients under Consideration for or Already in Active Surveillance: Correlation with Imaging Guided Target Biopsy. Diagnostics.

[10] Frantzi M., Hupe M.C., Merseburger A.S., Schanstra J.P., Mischak H., Latosinska A. (2020). Omics Derived Biomarkers and Novel Drug Targets for Improved Intervention in Advanced Prostate Cancer. Diagnostics.

[11] Saxby H., Mikropoulos C., Boussios S. (2020). An Update on the Prognostic and Predictive Serum Biomarkers in Metastatic Prostate Cancer. Diagnostics.

